# Stochastic oscillations and dragon king avalanches in self-organized quasi-critical systems

**DOI:** 10.1038/s41598-019-40473-1

**Published:** 2019-03-07

**Authors:** Osame Kinouchi, Ludmila Brochini, Ariadne A. Costa, João Guilherme Ferreira Campos, Mauro Copelli

**Affiliations:** 1 Universidade de São Paulo, Departamento de Física-FFCLRP, Ribeirão Preto, SP Brazil; 2 Universidade de São Paulo, Instituto de Matemática e Estatística, São Paulo, SP Brazil; 3 Universidade Federal de Goiás, Unidade Acadêmica Especial de Ciências Exatas, Jataí, GO Brazil; 4 Universidade Federal de Pernambuco, Departamento de Física, Recife, PE Brazil

## Abstract

In the last decade, several models with network adaptive mechanisms (link deletion-creation, dynamic synapses, dynamic gains) have been proposed as examples of self-organized criticality (SOC) to explain neuronal avalanches. However, all these systems present stochastic oscillations hovering around the critical region that are incompatible with standard SOC. Here we make a linear stability analysis of the mean field fixed points of two self-organized quasi-critical systems: a fully connected network of discrete time stochastic spiking neurons with firing rate adaptation produced by dynamic neuronal gains and an excitable cellular automata with depressing synapses. We find that the fixed point corresponds to a stable focus that loses stability at criticality. We argue that when this focus is close to become indifferent, demographic noise can elicit stochastic oscillations that frequently fall into the absorbing state. This mechanism interrupts the oscillations, producing both power law avalanches and dragon king events, which appear as bands of synchronized firings in raster plots. Our approach differs from standard SOC models in that it predicts the coexistence of these different types of neuronal activity.

## Introduction

Conservative self-organized critical (SOC) systems are by now well understood in the framework of out of equilibrium absorbing phase transitions^1–4^. But, since natural systems that present power law avalanches (earthquakes, forest fires etc.) are dissipative in the bulk, compensation (drive) mechanisms have been proposed to make the models at least conservative on average. However, it seems that these mechanisms do not work so well: they produce “dirty criticality”, in which the system hovers around the critical region with stochastic oscillations (SO). This behavior, characteristic of dissipative systems (say forest-fire models^5,6^ and the facilitated sandpile of^7^) has been called self-organized quasi-criticality (SOqC)^8,9^.

A subclass of systems that present SOqC behavior is the so called adaptive SOC (here aSOC) models and refers to networks that have an explicit dynamics in topology or parameters (link deletion and creation, adaptive synapses, adaptive gains)^10–15^. In the area of neuronal avalanches^16–19^, a well known aSOC system that presents SO is the Levina, Herrmann and Geisel (LHG) model^9,20,21^. This model uses continuous time leaky integrate-and-fire neurons in a complete graph topology and dynamic synapses with short-term depression inspired by the work of Tsodyks and Markram^22,23^. After that, similar models have been studied, for example excitable cellular automata^24^ in an Erdos-Renyi graph with LHG synapses^25,26^ and discrete time stochastic neurons in a complete graph with dynamic neuronal gains^14,15,27^.

Notice that not all systems that presents SOqC (forest-fire models^5,8^ for example) are aSOC systems (which always have adaptive network parameters) but it seems that all aSOC systems have SOqC behavior, with its characteristic stochastic oscillations hovering around the critical region. The exact nature of these stochastic oscillations is a bit unclear^8,9,15,27^. Here, we examine some representative discrete time aSOC models at the mean-field (MF) level. We find that they evolve as 2d MF maps whose fixed point is a stable focus very close to a Neimark-Sacker bifurcation, which defines the critical point. Recall that at the Neimark-Sacker bifurcation the focus loses its stability, turning into an indifferent centre with no attraction basin.

The MF map describes an infinite size system. For finite networks, finite size fluctuations (“demographic noise”) perturbs the almost unstable focus, fueling the SO. This kind of stochastic oscillation is known in the literature, sometimes called quasicycles^28–34^ but, to produce them, one ordinarily needs to fine tune the system close to the bifurcation point. In contrast, for aSOC systems, there is a self-organization dynamics that tunes the system very close to the critical point^9,14,15,20,25,26^. The SO orbits are sandwiched between the fixed point and the absorbing state, and frequently fall into the latter. This breaks the SO: we get not full oscillations, but a series of avalanches, some of then very large (dragon kings) and most of them small (with a power law size behavior).

Although aSOC models show no exact criticality, they are very interesting because they are the state-of-art models and can explain the coexistence of power law distributed avalanches and very large events (“dragon kings”^15,27,35–37^). Also, the adaptive mechanisms are biologically plausible and local, that is, they do not use non-local information to tune the system toward the critical region as occurs in other models^38,39^.

## Network Model with Stochastic Neurons

Our basic elements are discrete time stochastic integrate-and-fire neurons^40–44^. They enable simple and transparent analytic results^14,15^ but have not been intensively studied. We consider a fully connected topology with *i* = 1, …, *N* neurons. Let *X*_*i*_ be a firing indicator: *X*_*i*_[*t*] = 1 means that neuron *i* spiked at time *t* and *X*_*i*_[*t*] = 0 indicates that neuron *i* was silent at time *t*. Each neuron spikes with a firing probability function Φ(*V*_*i*_[*t*]) that depends on a real valued variable *V*_*i*_[*t*] (the membrane potential of neuron *i* at time *t*). Notice that, although the firing indicator is binary, the model is not a binary cellular automaton, but corresponds to a stochastic version of leaky integrate-and-fire neurons.

**Figure 1:**
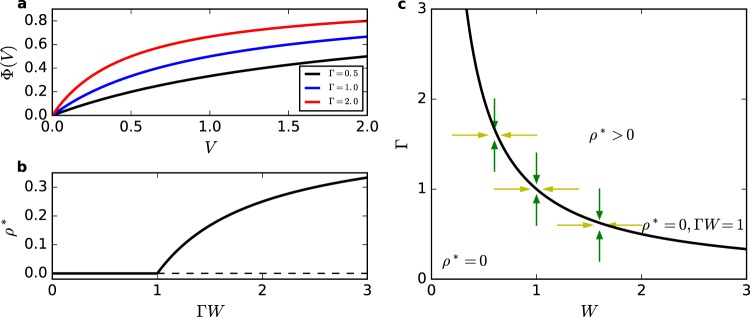
Firing function Φ(*V*), firing density and phase diagram for the static model. (**a**) Rational firing function Φ(*V*) for Γ = 0.5 (bottom), 1.0 (middle) and 2.0 (top). (**b**) Firing density ρ*(Γ*W*). The absorbing state *ρ*^0^ = 0 looses stability after Γ*W* > Γ_*c*_*W*_*c*_ = 1. (**c**) Phase diagram in the Γ × *W* plane. An aSOC network can be created by adapting synapses (horizontal arrows) or adapting neuronal gains (vertical arrows) toward the critical line.

The firing function can be any general monotonically increasing function 0 ≤ Φ(*V*) ≤ 1. For mathematical convenience, we use the so-called rational function^15^:

1 $$P({X}_{i}[t]=1|{V}_{i}[t])={\rm{\Phi }}({V}_{i}[t])=\frac{{\rm{\Gamma }}{V}_{i}[t]}{1+{\rm{\Gamma }}{V}_{i}[t]},$$

where Γ is the neuronal gain (the derivative *d*Φ/*dV* for small *V*). This firing function is shown in Fig. 1a.

In a general case the membrane voltage evolves as:

2 $${V}_{i}[t+\mathrm{1]}=\{\begin{array}{ll}\mu {V}_{i}[t]+{I}_{i}[t]+\frac{1}{N}\sum _{j=1}^{N}\,{W}_{ij}{X}_{j}[t] & \,{\rm{i}}{\rm{f}}\,{X}_{i}[t]=0,\\ 0 & \,{\rm{i}}{\rm{f}}\,{X}_{i}[t]=1\end{array}$$

where *μ* is a leakage parameter and *I*_*i*_[*t*] is an external input. The synaptic weights *W*_*ij*_ (*W*_*ii*_ = 0) are real valued with average *W* and finite variance. In the present case we study only excitatory neurons (*W*_*ij*_ > 0) but the model can be fully generalized to an excitatory-inhibitory network with a fraction *p* of excitatory and *q* = 1 − *p* inhibitory neurons^45^. The voltage is reset to zero after a firing event in the previous time step. Since Φ(0) = 0, this means that two consecutive firings are not allowed (the neuron has a refractory period of one time step).

In this paper we are interested in the second order absorbing phase transition that occurs when the external fields *I*_*i*_ are zero. Also, the universality class of the phase transition is the same for any value of *μ*^15^, so we focus our attention to the simplest case *μ* = 0. In the MF approximation, we substitute *X*_*i*_ by its mean value *ρ* = 〈*X*_*i*_〉, which is our order parameter (density of firing neurons or density of active sites). Then, Eq. (2) reads *V*[*t* + 1] = *Wρ*[*t*], where *W* = 〈*W*_*ij*_〉. From the definition of the firing function Eq. (1), we have:

3 $$\rho [t]=\int \,{\rm{\Phi }}(V)\,p(V)[t]\,dV,$$

where *p*(*V*)[*t*] is the voltage density at time *t*.

To proceed with the stability analysis of fixed points, it suffices to obtain the map for *ρ*[*t*] close to stationarity. The evolution of *ρ*[*t*] in the general case is thoroughly explored in Brochini *et al*. (2016)^14^ and Costa, Brochini and Kinouchi (2017)^15^. In the current case, *ρ*[*t*] evolves as follows. After a transient where all neurons spike at least one time, Eq. (2) lead to a voltage density that has two Dirac peaks, *p*(*V*)[*t* + 1] = *ρ*[*t*]*δ*(*V*) + (1 − *ρ*[*t*])*δ*(*V* − *Wρ*[*t*]). Inserting *p*(*V*) in Eq. (3), we finally get the 1d map:

4 $$\rho [t+1]=\frac{{\rm{\Gamma }}W\rho [t](1-\rho [t])}{1+{\rm{\Gamma }}W\rho [t]}.$$

The 1 − *ρ*[*t*] fraction corresponds to the density of silent neurons in the previous time step. We call this the static model because *W* and Γ are fixed control parameters.

The order parameter *ρ* has two fixed points: the absorbing state *ρ*^0^ = 0, which is stable (unstable) for Γ*W* < 1, (> 1) and a non-trivial firing state:

5 $${\rho }^{\ast }=\frac{{\rm{\Gamma }}W-1}{2{\rm{\Gamma }}W},$$

which is stable for Γ*W* > 1. This means that Γ*W* = 1 is a critical line of a continuous absorbing state transition (transcritical bifurcation), see Fig. 1b,c. If parameters are put at this critical line, one observes well behaved power laws for avalanche sizes and durations with the mean-field exponents −3/2 and −2 respectively^14^. However, such fine tuning should not be allowed for systems that are intended to be self-organized in criticality.

### Stochastic neurons model with dynamic synapses

Adaptive SOC models try to turn the critical point into an attractive fixed point of some homeostatic dynamics. For example, in the LHG model, the spike of the presynaptic neuron produces depression of the synapse, which recovers within some large time scale^9,20^.

A model with stochastic neurons and LHG synapses uses the same Eqs (1 and 2), but now the synapses change with time^14^:

6 $${W}_{ij}[t+1]={W}_{ij}[t]+\frac{{\rm{\Delta }}t}{\tau }(A-{W}_{ij}[t])-u{W}_{ij}[t]{X}_{j}[t].$$

Here, *τ* is the recovery time scale and 0 < *u*  < 1 is the fraction of synaptic strength that is lost when the presynaptic neuron fires (*X*_*j*_ = 1). From now on, we always use Δ*t* = 1 ms, the typical width of a spike.

### Stochastic neurons model with dynamic neuronal gains

Instead of adapting synapses toward the critical line *W*_*c*_ = 1/Γ, we can adapt the gains toward the critical condition Γ_*c*_ = 1/*W*, see Fig. 1c. This can be modeled as individual dynamic neuronal gains Γ_*i*_[*t*] (*i* = 1, …, *N*) that decrease by a factor *u* if the neuron fires (diminishing the probability of subsequent firings) with a recovery time 1/*τ* toward a baseline level *A*^14^:

7 $${{\rm{\Gamma }}}_{i}[t+1]={{\rm{\Gamma }}}_{i}[t]+\frac{1}{\tau }(A-{{\rm{\Gamma }}}_{i}[t])-u{\rm{\Gamma }}[t]{X}_{i}[t],$$

which is very similar to the LHG dynamics. Notice, however, that here we have only *N* equations for the neuronal gains instead of *N*(*N* − 1) equations for dynamic synapses, which allows the simulation of much larger systems. Moreover, the neuronal gain depression occurs due to the firing of the neuron *i* (that is, *X*_*i*_[*t*] = 1) instead of the firing of the presynaptic neuron *j*. The biological location is also different: adaption of neuronal gains (that produces firing rate adaptation) is a process that occurs at the axonal initial segment (AIS)^46,47^ instead of dendritic synapses.

Like the LHG model, this dynamics inconveniently has three parameters (*τ*, *A* and *u*). Recently, we proposed a simpler dynamics with only one parameter^15,27^:

8 $${{\rm{\Gamma }}}_{i}[t+1]={{\rm{\Gamma }}}_{i}[t]+\frac{1}{\tau }{{\rm{\Gamma }}}_{i}[t]-{{\rm{\Gamma }}}_{i}[t]{X}_{i}[t]=(1+\frac{1}{\tau }-{X}_{i}[t]){{\rm{\Gamma }}}_{i}[t].$$

Averaging over the sites, we obtain the 2d MF map:

9 $$\rho [t+1]=\frac{{\rm{\Gamma }}[t]W\rho [t](1-\rho [t])}{1+{\rm{\Gamma }}[t]W\rho [t]},$$

10 $${\rm{\Gamma }}[t+1]=(1+\frac{1}{\tau }-\rho [t])\,{\rm{\Gamma }}[t].$$

### Stability analysis for stochastic neurons with simplified neuronal gains

This case with a single-parameter dynamics (*τ*) for the neuronal gains has the simplest analytic results, so it will be presented first and with more detail. For finite *τ*, *ρ*^0^ = 0 is no longer a solution, see Eq. (10), and the 2d map has a single fixed point (ρ^*^, Γ^*^):

11 $${\rho }^{\ast }=\frac{1}{\tau },\,\,{{\rm{\Gamma }}}^{\ast }=\frac{{{\rm{\Gamma }}}_{c}}{1-\mathrm{2/}\tau },$$

where Γ_*c*_ = 1/*W*. The relation between ρ^*^ and Γ^*^ is:

12 $${\rho }^{\ast }=\frac{{{\rm{\Gamma }}}^{\ast }W-1}{2{{\rm{\Gamma }}}^{\ast }W}=\frac{{{\rm{\Gamma }}}^{\ast }-{{\rm{\Gamma }}}_{c}}{2{{\rm{\Gamma }}}^{\ast }},$$

which resembles the expression for the transcritical phase transition in the static system, see Eq. (5). Here, however, Γ^*^ is no longer a parameter to be tuned but rather a fixed point of the 2d map to which the system dynamically converges. Notice that the critical point of the static model can be approximated for large *τ*, with (ρ^*^, Γ^*^) → _*τ*→∞_(0, Γ_*c*_).

Performing a linear stability analysis of the fixed point (see Supplementary Information), we find that it corresponds to  a stable focus. The modulus of the complex eigenvalues is:

13 $$|{\lambda }^{\pm }|=\sqrt{1-\frac{\tau +2}{\tau (\tau -1)}}.$$

For large *τ* we have |*λ*^±^| = 1 − *O*(1/*τ*), with a Neimark-Sacker-like critical point occurring when |*λ*^±^| = 1 where the becomes indifferent. For example, we have |*λ*^±^| ≈ 0.990 for *τ* = 100, |*λ*^±^| ≈ 0.998 for *τ* = 500 and |*λ*^±^| ≈ 0.999 for *τ* = 1,000. Since, due to biological motivations, *τ* is in the interval of 100–1000 ms, we see that the focus is at the border of losing their stability. We call this point Neimark-Sacker-like because, in contrast to usual Neimark-Sacker one, the other side of the bifurcation, with |*λ*^±^| > 1, does not exist.

### Finite size fluctuations produce stochastic oscillations

The stability of the fixed point focus is a result for the MF map that represents an infinite system without fluctuations. However, fluctuations are present in any finite size system and these fluctuations perturb the almost indifferent focus, exciting and sustaining stochastic oscillations that hover around the fixed point.

**Figure 2:**
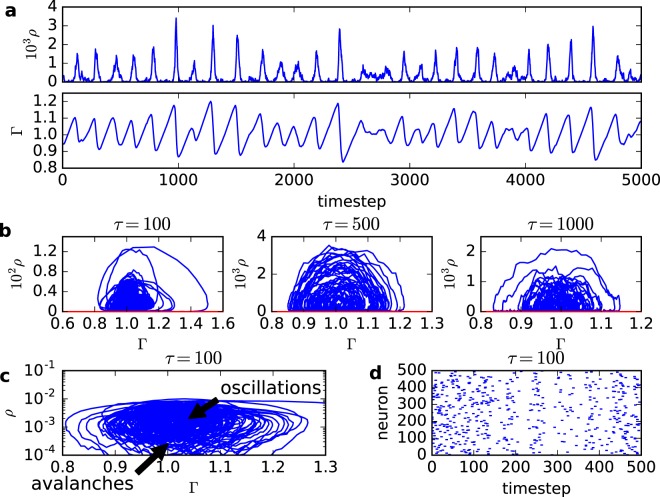
Stochastic neurons network with simplified (Eq. (8)) neuronal gain dynamics and *W* = 1. (**a**) Stochastic oscillations for *ρ*[*t*] and Γ[*t*] (*τ* = 500, *N* = 100,000 neurons). The quasiperiodic large events in the *ρ*[*t*] time series are the Dragon Kings and the small activity in between is composed of ordinary power law avalanches. (**b**) SO in the *ρ* vs Γ phase plane for *τ* = 100, 500 and 1,000 (*N* = 100,000). (**c**) SO in the log *ρ* vs Γ phase plane for *τ* = 100. (**d**) Raster plot with 500 neurons for *τ* = 100.

Without loss of generality, we fix *W* = 1 in the simulations for the simple model with one parameter dynamics defined by Eq. (8). In Fig. 2a, we show the SO for the firing density *ρ*[*t*] and average gain Γ[*t*] (*τ* = 500 and *N* = 100,000). As observed in the original LHG model^9^, the stochastic oscillations have a sawtooth profile with no constant amplitude or period. In Fig. 2b, we show the SO in the phase plane *ρ* vs Γ for *τ* = 100, 500 and 1,000.

For small amplitude (harmonic) oscillations, the frequency is given by (see Supplementary Information):

14 $$\omega =\arctan \frac{\sqrt{\tau +\mathrm{2/}\tau -4}}{\tau -2}.$$

The oscillation period, also for small amplitudes, is given by *T* = 2*π*/*ω*. The full oscillations are non-linear and their frequency and period depend on the amplitude.

Notice that, in the critical case, we have full critical slowing down: ${\mathrm{lim}}_{\tau \to \infty }\omega ={\tau }^{-1/2}\to 0,{\mathrm{lim}}_{\tau \to \infty }T\to \infty$. This means that, exactly at the critical point, the 2d map corresponds to a center without a period, and the SO would correspond to critical fluctuations similar to random walks in the *ρ* vs Γ plane. However, since for any physical/biological system the recovery time *τ* is finite, the critical point is not observable.

### Stochastic oscillations and avalanches

For large *τ*, part of the SO orbit occurs very close to the zero state *ρ*^0^ = 0, see Fig. 2b,c. Due to finite-size fluctuations, the system frequently falls into this absorbing state. The orbits in phase space are interrupted. As in usual SOC simulations, we can define the size of an avalanche as the number *S* of firing events between such zero states. After a zero state, we force a neuron to fire, to continue the dynamics, so that Fig. 2b,c are better understood as a series of patches (avalanches) terminating at *ρ*[*t*] = 0, not as a single orbit.

**Figure 3:**
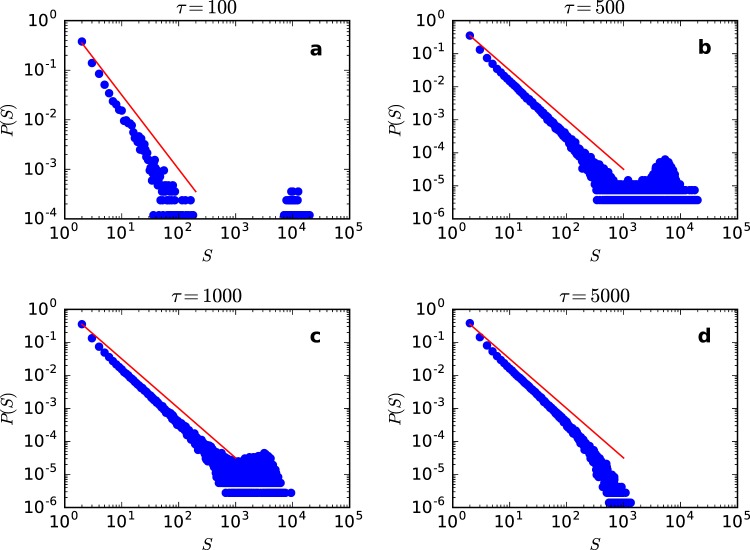
Avalanche size distribution *P*(*S*) for the simplified neuronal gain dynamics, Eq. (8). (**a**) *τ* = 100. (**b**) *τ* = 500. (**c**) *τ* = 1,000 and (**d**) *τ* = 5,000. All plots have *N* = 100,000 and *W* = 1. The straight line corresponds to the exponent −3/2. The apparent subcriticality in **d** is a finite size effect.

The presence of the SO affects the distribution of avalanche sizes *P*(*S*), see Fig. 3. For small *τ*, ρ^*^ is larger and it is more difficult for the SO to fall into the absorbing state. We observe a bump of very large avalanches (dragon king events). Increasing *τ*, we move closer to the Neimark-Sacker-like critical point and observe power law avalanches with exponent −3/2 similar to those produced in the static model that suffers a transcritical bifurcation. So, by using a different mechanism (Neimar-Sacker-like versus transcritical bifurcation), our aSOC model can reproduce experimental data about power law neuronal avalanches^16–19^. It also predicts that these avalanches can coexist with dragon kings events^36,37^.

The *P*(*S*) distribution is not our main concern here and a more rigorous finite size analysis can be found in previous papers^14,15^.

Notice that the dragon kings in Fig. 3a–c have size up to 10^4^, that is, involve 10% of the whole network. This is a large fraction for an avalanche. After a dragon king, the depressive activity dependent mechanism produces an abrupt fall of the average Γ[*t*], see Fig. 2 second panel. That is, most of the smaller avalanches occurs when Γ[*t*] is below the critical value Γ_*c*_ = 1: effectively, they are subcritical avalanches, with slightly subcritical power laws, as we can see when compared to the critical (red line) −3/2 power law.

In the case of Fig. 3d we have no dragon kings, with *τ* = 5000 seeming to be subcritical. In fact, this is due to a finite size effect because, the larger the *τ*, the larger the *N* value needs to be in order to produce a critical Γ close enough to the Γ^*^ in the thermodynamic limit (see the sigmoidal behavior in Fig. 5 of^15^).

### Stochastic neurons with LHG dynamic gains

We now return to the case of dynamic gains with LHG dynamics, see Eq. (7). Without loss of generality, we use *W* = 1, so that the static model has Γ_*c*_ = 1/*W* = 1. The map is given by:

15 $$\rho [t+1]=\frac{{\rm{\Gamma }}[t]\rho [t](1-\rho [t])}{1+{\rm{\Gamma }}[t]\rho [t]},$$

16 $${\rm{\Gamma }}[t+1]={\rm{\Gamma }}[t]+\frac{1}{\tau }(A-{\rm{\Gamma }}[t])-u\,{\rm{\Gamma }}[t]\rho [t].$$

The trivial absorbing fixed point is (*ρ*^0^, Γ^0^) = (0, *A*), stable for *A* < 1, see Supplementary Material. For *A* > 1, we have a non-trivial fixed point given by:

17 $${\rho }^{\ast }=\frac{A-1}{2A+\tau u},$$

18 $${{\rm{\Gamma }}}^{\ast }=\frac{2A+\tau u}{2+\tau u}={{\rm{\Gamma }}}_{c}+\frac{2(A-\mathrm{1)}}{2+\tau u}.$$

There is a relation between ρ^*^ and Γ^*^ that resembles the relation between the order parameter and the control parameter in the static model:

19 $${\rho }^{\ast }=\frac{{{\rm{\Gamma }}}^{\ast }-1}{2{{\rm{\Gamma }}}^{\ast }},$$

valid for Γ^*^ > Γ_*c*_ = 1. Here, however, Γ^*^ is a self^-^organized variable, not a control parameter to be finely tuned.

We notice that to set by hand *A* = 1, pulling all gains toward Γ_*i*_ = 1, produces the critical point (ρ^*^, Γ^*^) = (0, 1). Nevertheless, this is a fine tune that should not be allowed for SOC systems. We must use *A* > 1, and reach the critical region only at the large *τ* limit: ρ^*^ ≈ (*A* − 1)/*τu* and Γ^*^ ≈ Γ_*c*_ + 2(*A* − 1)/*τu*.

Proceeding with the linear stability analysis, we obtain (see Supplementary Information):

20 $${\lambda }^{+}{\lambda }^{-}=(1-\frac{1}{\tau })(1-\frac{2(A-1)}{A+u\tau +1})+\frac{u{(A-1)}^{2}}{(A+u\tau +1)(2A+u\tau )}.$$

To first order in *τ*, we have:

21 $$|{\lambda }^{\pm }|=\sqrt{{\lambda }^{+}{\lambda }^{-}}=1-\frac{2(A-1)+u}{2u\tau }+O({\tau }^{-2}),$$

which means that, for large *τ*, the map is very close to the Neimark-Sacker-like critical point. As the stable focus approaches the critical value, the system exhibits oscillations as it approaches the fixed point due to demographic noise, leading to SO in finite-sized systems.

**Figure 4:**
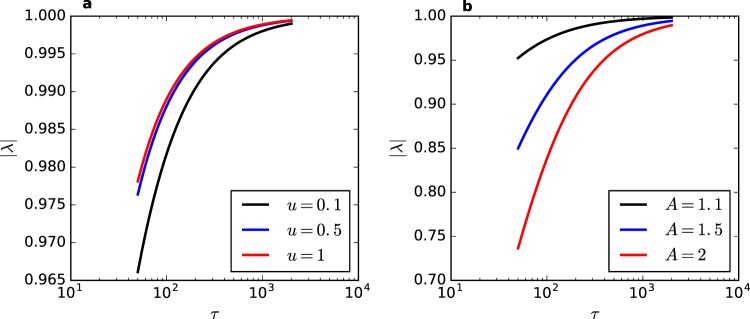
Modulus |*λ*^±^| as a function of *τ* for several values of *u* and *A*. (**a**) From top to bottom, *u* = 0.1, 0.5 and 1.0, for *A* = 1.05. (**b**) From top to bottom, *A* = 1.1, 1.5 and *A* = 2.0, for *u* = 0.1. For these values of *A* and *u*, the difference between exact *λ* values and the first order approximation in Eq. (21) are at most 1% for *τ* ≥ 100.

For example, with the typical values *A* = 1.05 and *u* = 0.1^25,26^, we have |*λ*^±^| ≈ 1 − 1/*τ*, which gives |*λ*^±^| ≈ 0.990 for *τ* = 100 and |*λ*^±^| ≈ 0.999 for *τ* = 1,000. In Fig. 4, we present the exact |*λ*^±^|, the square root of Eq. (20), as a function of *τ* for several values of *A* and *u*. For large *τ*, the frequency for harmonic oscillations is given by $\omega \simeq \sqrt{(A-\mathrm{1)/}\tau }$ (see Supplementary Information).

The analysis of the stochastic neuron model with LHG synapses *W*_*ij*_[*t*], see Eq. (6), is very similar to the one above with LHG dynamic gains Γ_*i*_[*t*]. The only difference is that we need to exchange Γ by *W*. The eigenvalue modulus is the same, so that LHG dynamic synapses also produce SO. But, instead of presenting simulations in our complete graph system, which would involve *N*(*N* − 1) dynamic equations for the synapses, we prefer to discuss the LHG synapses for another system well known in the literature. We will also examine how SO depends on the system size *N*.

## Excitable Cellular Automata with LHG Synapses

We now consider an aSOC version of a probabilistic cellular automata well studied in the literature^24,48–52^. It is a model for excitable media that yields a natural interpretation in terms of neuronal networks. Each site has *n* states, *X* = 0 (silent), *X* = 1 (firing), *X* = 2, …, *n* − 1 (refractory). Here we will use *n* = 2.

Each neuron *i* = 1, …, *N* has *K*_*i*_ random neighbours (the average number in the network is *K* = 〈*K*_*i*_〉) coupled by probabilistic synapses *P*_*ij*_∈[0, 1] (here we use *K* = 10, for implementation details see^24–26^). If the presynaptic neuron *j* fires at time *t*, with probability *P*_*ij*_ the postsynaptic neuron *i* fires at *t* + 1. The update is done in parallel, so the synapses are multiplicative, not additive like in the stochastic neuron model. All neurons that fire are silenced in the next step. Only neurons in the silent state can be induced to fire. Since 1 − *P*_*ij*_*X*_*j*_ is the probability that neighbour *j* does not induce the firing of neuron *i*, we can write the update rule as:

22 $$P({X}_{i}[t+1]=1)=(1-{X}_{i}[t])[1-\prod _{j}^{{K}_{i}}\,(1-{P}_{ij}{X}_{j})],$$

The control parameter of the static model is the branching ratio *σ* = *K*〈*P*_*ij*_〉 and the critical value is *σ*_*c*_ = 1.

In the cellular automata (CA) model with LHG synapses, we have^25,26^:

23 $${P}_{ij}[t+\mathrm{1]}={P}_{ij}[t]+\frac{1}{\tau }(\frac{A}{K}-{P}_{ij}[t])-u{P}_{ij}[t]{X}_{j}[t].$$

### Mean field stability analysis

The 2d MF map close to the stationary state is:

24 $$\rho [t+1]=(1-\rho [t])[1-{(1-\frac{\sigma [t]\rho [t]}{K})}^{K}],$$

25 $$\sigma [t+1]=\sigma [t]+\frac{1}{\tau }(A-\sigma [t])-u\sigma [t]\rho [t],$$

where in the second line we multiplied Eq. (23) by *K* and averaged over the synapses.

There is a trivial absorbing fixed point (*ρ*^0^, *σ*^0^) = (0, *A*), stable up to *A* = 1, see Supplementary Material. For *A* > 1, there exists a single stable fixed point given implicitly by:

26 $${\rho }^{\ast }=(1-{\rho }^{\ast })[1-{(1-\frac{A{\rho }^{\ast }}{(1+u\tau {\rho }^{\ast })K})}^{K}],$$

27 $${\sigma }^{\ast }=\frac{A}{1+u\tau {\rho }^{\ast }},$$

which we can find numerically.

For this model we have numerical but no analytic results. However, we can find an approximate solution close to criticality, where ρ^*^ is small (see Supplementary Information). Expanding in powers of 1/*τ*, we get:

28 $$|{\lambda }^{\pm }|=1-(\frac{(A-1)(2K-1)}{2uK}+\frac{1}{2})\,\frac{1}{\tau }+O({\tau }^{-2}).$$

This confirms that the same scenario of a weakly stable focus appears in the CA model.

This last result is particularly interesting. If we put *K* = *N* − 1, the CA network becomes a complete graph. Performing the limit *N* → ∞, Eq. (28) gives:

29 $$|{\lambda }^{\pm }|=1-\frac{A-1+u/2}{u\tau }+O({\tau }^{-2}),$$

which is identical to the |*λ*^±^| value for the LHG stochastic neuron model, see Eq. (21).

Concerning the simulations, we must make a technical observation. In contrast to the static model^24^, the relevant indicator of criticality here is no longer the branching ratio *σ*, but the principal eigenvalue Λ ≠ *σ* of the synaptic matrix *P*_*ij*_, with Λ_*c*_ = 1^48,51^. This occurs because the synaptic dynamics creates correlations in the random neighbour network^26^. Therefore, the MF analysis, where correlations are disregarded, does not furnish the exact *σ* or Λ of the CA model. But there exists an annealed version of the model in which σ^*^ = Λ^*^ and the MF analysis fully holds^25,26^.

In this annealed version, when some neuron fires, the depressing term −*uP*_*ij*_ is applied to *K* synapses randomly chosen in the network. This is not biologically realistic, but destroys correlations and restores the MF character of the model. Since all our analyses are done at the MF level, we prefer to present the simulation results using the annealed model. Concerning the SO phenomenology, there is no qualitative difference between the annealed and the original model defined by Eq. (23).

**Figure 5:**
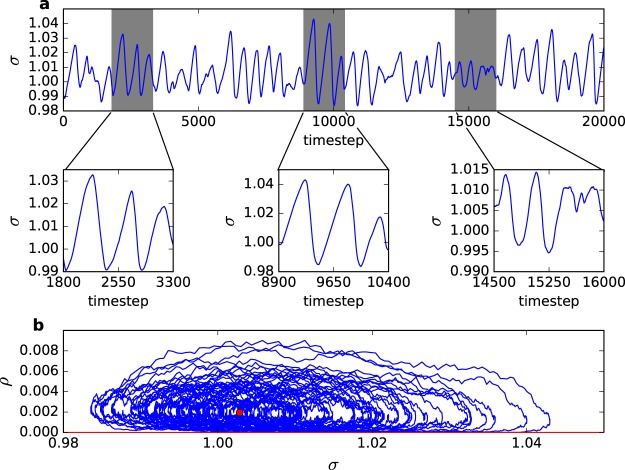
Stochastic oscillations for the annealed CA model with LHG synapses. (**a**) The SO for *σ*[*t*] has a sawtooth profile where large amplitudes have low frequency (*N* = 128,000 neurons, *τ* = 500, *A* = 1.1 and *u* = 0.1). (**b**) The SO in the phase plane *ρ* vs *σ*. The red bullet is the fixed point given by Eqs (26 and 27).

**Figure 6:**
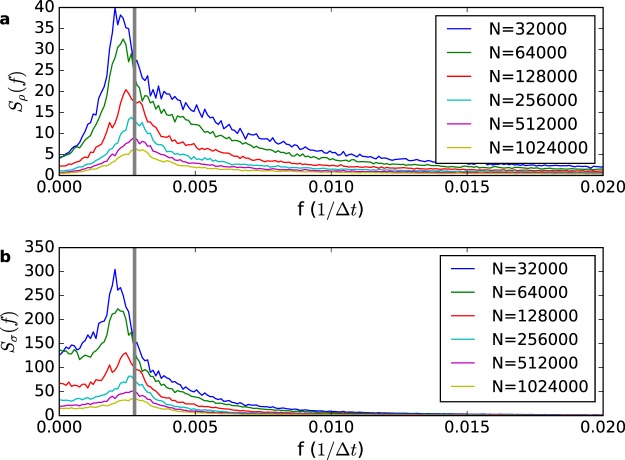
Power spectrum of the time evolution of *ρ* and *σ* in the annealed CA model with LHG synapses. (**a**,**b**), power spectrum of the time evolution of *ρ* and *σ*, respectively, for different system sizes (*τ* = 320, *A* = 1.1, *u* = 0.1). The vertical lines mark the theoretical value of the frequency of small oscillations of the evolution of the MF map near the fixed point calculated from Supplementary Information Eq. (69).

The full stochastic oscillations in a system with *N* = 128,000 neurons and *τ* = 500 can be seen in Fig. 5a,b. The angular frequency for small amplitude oscillations is given by Supplementary Information Eq. (69). We have $\omega \simeq \sqrt{(A-\mathrm{1)/}\tau }$ for large *τ*, which is the same behavior found for stochastic neurons. The power spectrum of the time evolution of *ρ* and *σ* is shown in Fig. 6. The peak frequency gets closer to the theoretical *ω* (vertical line) for larger system sizes because the oscillations have smaller amplitudes, going to the small oscillations limit.

### Dependence of the stochastic oscillations on system size

Now we ask if the SO survive in the thermodynamic limit. In principle, given that fluctuations vanish in this limit, and we have always damped focus for finite *τ*, the SO should also disappear when 1/*N* → 0. In contrast, Bonachela *et al*. claimed that, in the LHG model, the amplitude of the oscillations basically does not change with *N* and is non-zero in the thermodynamic limit^9^. Indeed, they proposed that this feature is a core ingredient of self-organized quasi-criticality.

**Figure 7:**
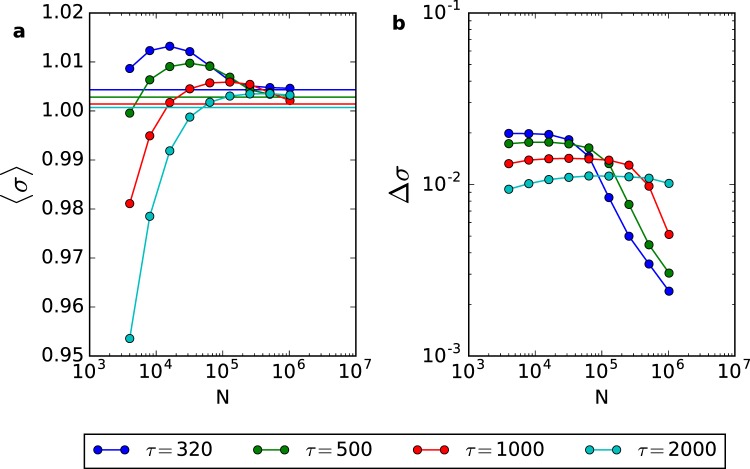
Average and standard deviation of the SO time series (CA model) as a function of *N*. (**a**) Average 〈*σ*[*t*]〉 for different *τ* values. Horizontal lines are the value of the fixed points σ^*^ given by Eq. (27). (**b**) Standard deviation Δ*σ* for different *τ* values. Parameters are *A* = 1.1 and *u* = 0.1. All measures are taken in a window of 10^6^ time steps after discarding transients.

In Fig. 7, we measured the average 〈*σ*[*t*]〉 and the standard deviation Δ*σ* of the *σ*[*t*] time series of the annealed model. We used *τ* = 320, 500, 1,000 and 2,000 and system sizes from *N* = 4,000 to 1,024,000. We interpret our findings as a *τ* dependent crossover phenomenon due to a trade-off between the level of fluctuations (which depends on *N*) and the level of dampening (which depends on *τ*): for a given *τ*, a small *N* can produce sufficient fluctuations so that the SO are sustained without change of Δ*σ*. Nonetheless, starting from some *N*(*τ*), the fluctuations are not sufficient to compensate the dampening given by |*λ*^±^| < 1 and Δ*σ* starts to decrease for increasing *N*. The larger the *τ*, the less damped is the focus and the SO survive without change to a larger *N* (the plateau in Fig. 7b is more extended to the right).

So, the conclusion is that, for any finite *τ*, the SO do not survive up to the thermodynamic limit, although they can be observed in very large systems. This is compatible with Fig. 6 where the power spectrum decreases with *N*. We also see in Fig. 7a that 〈*σ*[*t*]〉 → σ^*^ for increasing *N*, so the system settles without variance at the MF fixed point (ρ^*^, σ^*^) in the infinite size limit.

We can reconcile our findings with those of the LHG model^9,20^ remembering an important technical detail: these authors used a synaptic dynamics with *τ* = *τ*_0_*N*, in an attempt to have the fixed point converge to the critical one in the thermodynamic limit (the same scaling is used in other models^13,25^). In systems with this scaling, the fluctuations decay with *N* but, at the same time, the dampening controlled by *λ*(*τ*(*N*)) also decays with *N*. For this scaling, we can accord that the SO survive in the thermodynamic limit. However, we already emphasized that this is a non-biological and non-local scaling choice^14,15,26^ because the recovery time *τ* must be finite and the knowledge of the network size *N* is non-local.

However, from a biological point of view, this discussion is not so relevant: although the finite-size fluctuations (called demographic noise in the literature^29,30,33,34^) disappear for large *N*, external (environmental) noise of biological origin, not included in the models, never vanishes in the thermodynamic limit. So, for practical purposes, the SO and the associated dragon kings would always be present in more realistic noisy networks and experiments.

## Discussion

We now must stress what is new in our findings. Standard SOC models are related to static systems presenting an absorbing state phase transition^1–4^. At the MF level, these static systems are described by a 1d map *ρ*[*t* + 1] = *F*(*ρ*[*t*]) for the density of active sites, and the phase transition corresponds to a transcritical bifurcation where the critical point is an indifferent node. This indifferent equilibrium enables the occurrence of scale-invariant fluctuations in the *ρ*[*t*] variable, that is, scale-invariant avalanches but not stochastic oscillations.

In aSOC networks, the original control parameter of the static system turns out to be an activity dependent variable leading to 2d MF maps. We performed a MF fixed point stability analysis for three systems: 1) a stochastic neuronal model with one-parameter neuronal gain dynamics, 2) the same model with LHG gain dynamics, and 3) the CA model with LHG synapses. For the first one we obtained very simple and transparent analytic results; for the LHG dynamics we also got analytic, although more complex, results; finally, for the CA model, we were able to obtain a first order approximation for large recovery time *τ*. Curiously, in the limit of *K* = (*N* − 1) → ∞ neighbours, the stochastic neuron model and the CA model have exactly the same first order leading term. The complex eigenvalues have modulus |*λ*^±^| ≈ 1 − *O*(1/*τ*) and, for large *τ*, the fixed point is a focus at the border of indifference. This means that, in finite size systems, the dampening is very low and fluctuations can excite and sustain stochastic oscillations.

About the generality of our results, we conjecture they are generic and valid for the whole class of aSOC models, from networks with discrete deletion-recovery of links^11–13^ to continuous depressing-recovering synapses^9,20,25,26^ and neurons with firing rate adaptation^14,15,27^). At the mean-field level, all of them are described by similar two-dimensional dynamical systems: one variable for the order parameter, another for the adaptive mechanism. For example, the prototypical LHG model^9,20^ uses continuous time LIF neurons (which is equivalent to setting Γ → ∞ and *μ* > 0 in our model). Although with more involved calculations, in principle one could do a similar mean-field calculation and obtain a 2d dynamical system with an almost unstable focus close to criticality, explaining the SO observed in that model. In another aspect of generality, stochastic oscillations have been observed previously in other self-organized quasi-critical systems (forest-fire models^5,6^ and some special sandpile models^7,53^) that share a similar MF description with aSOC systems. Indeed, it seems that stochastic oscillations are a distinctive feature of self-organized quasi-criticality, as defined by Bonachela *et al*.^8,9^.

We also emphasize that the fact that our network has only excitatory neurons is not a limitation of this study. In a future work^45^, we will show that our model can be fully generalized to an excitatory-inhibitory network very similar to the Brunel model^54^, with the same results.

So, it must be clear that what is new here is not the stochastic oscillations (quasicycles) in biosystems, since there is a whole literature about that^28–34^. What is new here is the interaction of the SO with a critical point with an absorbing state. This interaction interrupts the oscillations, producing the phenomenology of avalanches and dragon kings. This is our novel proposal for a mechanism that produces dragon kings coexisting with limited power law avalanches.

In conclusion, contrasting to standard SOC, aSOC systems present stochastic oscillations that will not vanish in the thermodynamic limit if external (environmental) noise is present. But what seems to be a shortcoming for neural aSOC models could turn out to be an advantage. Since the adaptive dynamics with large *τ* has good biological motivation, it is possible that SO are experimentally observable, providing new physics beyond the standard model for SOC. The presence of the Neimark-Sacker-like bifurcation affects the distribution of avalanche sizes *P*(*S*), creates quasi-periodic dragon king events, and all this phenomenology can be measured^35,55,56^. In particular, our raster plots results are compatible with system-sized events (synchronized fires) recently observed in neuronal cultures^36,37^. So, we have experimental predictions that differ from standard SOC based on a transcritical bifurcation (and also from criticality models with Griffiths phases^57,58^). We propose that the experimental detection of quasi-periodic dragon-kings coexisting with power law avalanches for small events could be the next experimental challenge in the field of neuronal criticality.

Finally, we speculate that non-mean field models (square or cubic lattices) with dynamic gains and SO could be applied to the modeling of dragon-kings (large quasiperiodic “characteristic”) earthquakes that coexist with power-law Gutemberg-Richter distributions for the small events^59,60^. This will be pursued in another work.

## Methods

### Numerical Calculations

Numerical calculations were done by using MATLAB softwares.

### Simulation procedures

Simulation codes were made in Fortran90 and C++11.

## Supplementary information

Supplementary information
