# Supplementary material for: Stochastic oscillations and dragon king avalanches in self-organized quasi-critical systems
Source: Sci Rep. 2019 Mar 7;9:3874. doi: 10.1038/s41598-019-40473-1 (PMC6405991; doi:10.1038/s41598-019-40473-1)
Supplement: Supplementary file 1 — Supplementary information [file 41598_2019_40473_MOESM1_ESM.pdf]

Supplementary Information of "Stochastic oscillations and dragon king avalanches in self-organized quasi-critical systems"

Osame Kinouchi, Ludmila Brochini, Ariadne A. Costa, João Guilherme Ferreira Campos, Mauro Copelli

Stability analysis of the stochastic neuron model with one parameter (τ) gain dynamics

The MF map is:

$$\rho[t+1] = \frac{\Gamma[t]W\rho[t](1-\rho[t])}{1+\Gamma[t]W\rho[t]} = F(\rho[t],\Gamma[t]) \,, \tag{1}$$

$$\Gamma[t+1] = \left(1+\frac{1}{\tau}-\rho[t]\right)\Gamma[t] = G(\rho[t],\Gamma[t]) \,. \tag{2}$$

Notice that $(\rho^0=0,\Gamma)$ is not a fixed point. The single fixed point is:

$$\rho^* = \frac{1}{\tau}\,, \qquad \Gamma^* = \frac{\Gamma_c}{1-2/\tau} \,. \tag{3}$$

To perform the fixed point stability analysis we calculate partial derivatives evaluated at the fixed point (denoted by $]_*$):

$$a = \left.\frac{\partial F}{\partial \rho}\right]_* = \frac{\Gamma^*W - 2\Gamma^*W\rho^* - (\Gamma^*W\rho^*)^2}{[1+\Gamma^*W\rho^*]^2} = \frac{\tau-3}{\tau-1}\,, \tag{4}$$

$$b = \left.\frac{\partial F}{\partial \Gamma}\right]_* = \frac{W\rho^*(1-\rho^*)}{[1+\Gamma^*W\rho^*]^2} = \frac{W(\tau-2)^2}{\tau^2(\tau-1)}\,, \tag{5}$$

$$c = \left.\frac{\partial G}{\partial \rho}\right]_* = -\Gamma^* = -\frac{\tau}{W(\tau-2)}\,, \tag{6}$$

$$d = \left.\frac{\partial G}{\partial \Gamma}\right]_* = 1+1/\tau-\rho^* = 1 \,. \tag{7}$$

The eigenvalues of the system Jacobian matrix at the fixed point (ρ^*,Γ^*) are determined by

$$\det(J-\lambda I) = (a-\lambda)(d-\lambda) - bc = 0 \,. \tag{8}$$

So, the characteristic equation is:

$$\lambda^2 - \left[\left(\frac{\tau-3}{\tau-1}\right)+1\right]\lambda + \frac{\tau-3}{\tau-1} + \frac{\tau-2}{\tau(\tau-1)} = 0 \,. \tag{9}$$

That can be written as:

$$\tau(\tau-1)\lambda^2 - (2\tau^2-4\tau)\lambda + \tau^2 - 2\tau - 2 = 0 \,, \tag{10}$$

with solutions:

$$\lambda^{\pm} = \frac{\tau^2-2\tau\pm\sqrt{\Delta}}{\tau(\tau-1)}\,, \tag{11}$$

$$\Delta = (\tau^2-2\tau)^2 - \tau(\tau-1)(\tau^2-2\tau-2) \tag{12}$$

This equation has no meaning for $\tau<2$ since this would give $\rho^* = 1/\tau > 1/2$. When $2<\tau<2+\sqrt{2}$ we have $\Delta>0$, λ real and $|\lambda|<1$, that is, the fixed point (ρ^*,Γ^*) is a stable node. However, when $\tau > 2+\sqrt{2} = 3.4142\ldots$, we have $\Delta<1$ and two complex roots $\lambda^{\pm}$ with $|\lambda| = \sqrt{\lambda^+\lambda^-} < 1$, that is, we have a stable focus with:

$$\lambda^{\pm} = \frac{\tau^2-2\tau\pm i\sqrt{-\Delta}}{\tau(\tau-1)}\,, \tag{13}$$

$$|\lambda^{\pm}| = \sqrt{\lambda^+\lambda^-} = \sqrt{\frac{\tau^2-2\tau-2}{\tau(\tau-1)}} = \sqrt{1-\frac{\tau+2}{\tau(\tau-1)}}\,, \tag{14}$$

which leads to Eq. (13) of the main Manuscript

For small amplitude (harmonic) oscillations, the frequency is given by the angle of the eigenvalues written in polar coordinates. We can write it in terms of the determinant D and trace T of the Jacobian matrix J as

$$\omega \equiv |\omega^{\pm}| = \arctan\sqrt{\frac{4D}{T^2}-1} = \arctan\frac{\sqrt{\tau+2/\tau-4}}{\tau-2} \tag{15}$$

When $\tau\to\infty$,

$$\omega \simeq \sqrt{\frac{1}{\tau}} \tag{16}$$

Case $\mu > 0$

In the case $\mu > 0$, instead of a single equation to describe $\rho[t]$, we need some recurrence equations to describe the evolution of firing densities. As described in[1] and[2], the density of firing neurons $\rho[t]$ can be computed from the probability density $p[t](V)$ of potentials at time t:

$$\rho[t] = \int_{-\infty}^{\infty}\Phi(V)p[t](V)\,dV\,, \tag{17}$$

Suppose that at time t_0 every neuron in the network has already fired at least once. Then, we can describe $\rho[t]$ for $t > T_0$ with respect to the evolution of densities of neurons according their firing age. In this case, Eq. (17) becomes:

$$\rho[t] = \sum_{k=1}^{\infty}\eta_k[t]\Phi(U_k[t])\,, \tag{18}$$

Where $\eta_k[t]$ is the proportion of neurons at time $t > T_0$ that fired k time steps ago, which all have potential $U_k[t]$. The proportion of neurons with firing age k at t corresponds to fraction of the population with firing age $k-1$ that did not fire at time $t-1$:

$$\eta_k[t] = \eta_{k-1}[t-1]\,(1-\Phi(U_{k-1}[t-1]))\,, \tag{19}$$

And from Eq. (2) of the main Manuscript we know that the potential evolution of these populations follow:

$$U_k[t] = \mu U_{k-1}[t] + W\rho[t]\,, \tag{20}$$

The normalization condition $\sum_{k=0}^{\infty}\eta_k[t] = 1$ must be included explicitly. Although, we cannot obtain a simple 2d map for ρ and Γ as is the case for $\mu = 0$, we can instead, solve Eqs (19–20) numerically for any μ at every time step as Γ evolves concomitantly according to Eq. (2).

In[2] we estimate from Eqs (19–20), approximating for small ρ, that in the stationary state:

$$\rho^* \approx \frac{1}{2+\mu+\mu^2/(1-\mu)}\,\frac{\Gamma-\Gamma_c}{\Gamma}\,. \tag{21}$$

where $\Gamma_c = (1-\mu)/W$.

Eq. (2) the value of ρ for the non trivial fixed point. Using its value in 21, we obtain that, for $\mu > 0$:

$$\rho^* = \frac{1}{\tau}\,, \qquad \Gamma^* \approx \frac{\Gamma_c}{1-\frac{1}{\tau C_\mu}} \tag{22}$$

where $C_\mu = 2+\mu+\mu^2/(1-\mu)$.

Figure 1 shows the numerical iteration for the simplified adaptive model with one parameter in two cases: for $\mu = 0$ (Fig. 1a), which corresponds to the 2D map in Eqs (1 and 2), and for $\mu = 0.5$ (Fig.~1b) by the iterations of Eqs (2, 19 and 20). Fig. 1b illustrates how the behavior of the system around the fixed point for $\mu > 0$ (Eqs (22), red dot) is qualitatively similar to the case $\mu = 0$.

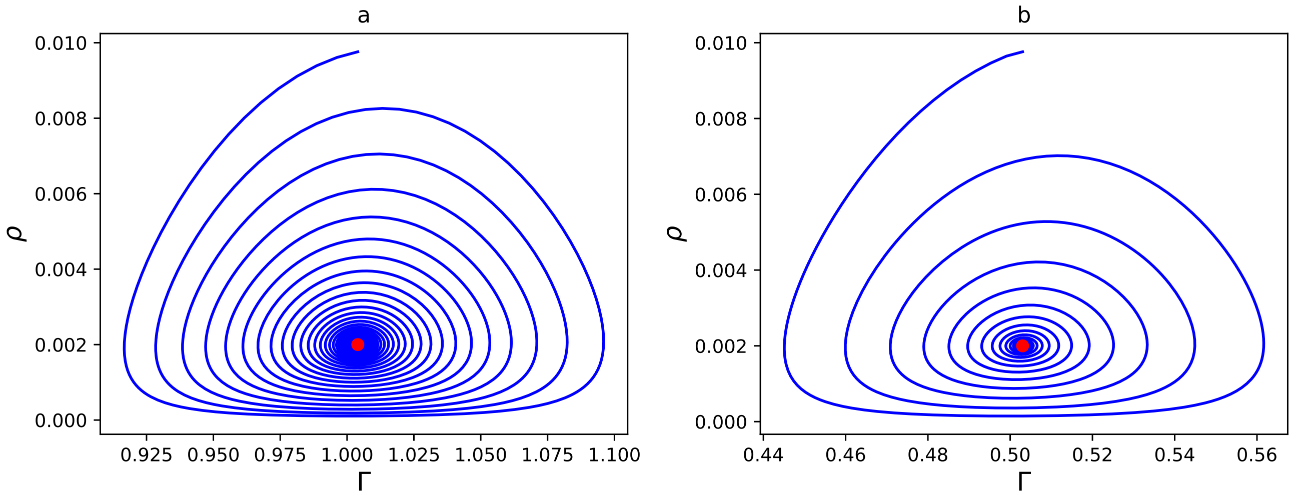

Figure 1. Example of transient behavior close to the fixed points.
a, Iteration of the MF map, given by Eqs (1 and 2) ($\mu = 0$). The fixed point depicted in red is a stable focus given by Eqs (3).
b, Evolution of ρ, Γ according to Eqs (2, (19 and 20) ($\mu = 0.5$). Fixed point depicted in red is given by Eq. (22). Parameter $\tau = 500$ in both cases.

Stability analysis of the stochastic neuron model with LHG gain dynamics

The MF map is given by:

$$\rho[t+1] = \frac{\Gamma[t]\rho[t](1-\rho[t])}{1+\Gamma[t]\rho[t]} = F(\rho[t], \Gamma[t]) \,, \tag{23}$$

$$\Gamma[t+1] = \Gamma[t] + \frac{1}{\tau}(A - \Gamma[t]) - u\Gamma[t]\rho[t] = G(\rho[t], \Gamma[t]) \,. \tag{24}$$

We have two fixed points. The absorbing state fixed point:

$$\rho^0 = 0 \qquad \Gamma^0 = A \,, \tag{25}$$

and the non trivial fixed point (ρ^*, Γ^*):

$$\rho^* = \frac{A-1}{2A+\tau u} \,, \qquad \Gamma^* = \frac{2A+\tau u}{2+\tau u} \,. \tag{26}$$

Partial derivatives of F and G with respect to ρ and Γ are:

$$\frac{\partial F(\rho,\Gamma)}{\partial \rho} = -\frac{\Gamma\left(2\rho + \rho^2\Gamma - 1\right)}{(\rho\Gamma+1)^2} \,, \tag{27}$$

$$\frac{\partial F(\rho,\Gamma)}{\partial \Gamma} = -\frac{(\rho-1)\rho}{(\rho\Gamma+1)^2} \,, \tag{28}$$

$$\frac{\partial G(\rho,\Gamma)}{\partial \rho} = -\Gamma u \,, \tag{29}$$

$$\frac{\partial G(\rho,\Gamma)}{\partial \Gamma} = -\frac{1}{\tau} - u\rho + 1 \,. \tag{30}$$

Evaluating at the trivial fixed point, we get:

$$a = \left.\frac{\partial F}{\partial \rho}\right]_0 = \Gamma = A\,, \tag{31}$$

$$b = \left.\frac{\partial F}{\partial \Gamma}\right]_0 = 0\,, \tag{32}$$

$$c = \left.\frac{\partial G}{\partial \rho}\right]_0 = -u\Gamma = -uA\,, \tag{33}$$

$$d = \left.\frac{\partial G}{\partial \Gamma}\right]_0 = 1 - \frac{1}{\tau}\,. \tag{34}$$

The eigenvalues are found from $(a-\lambda)(d-\lambda) - bc = 0$, or:

$$(A-\lambda)(1-1/\tau-\lambda) = 0\,, \tag{35}$$

$$\lambda^2 - (A+1-1/\tau)\lambda + (1-1/\tau)A = 0\,, \tag{36}$$

with solution:

$$\lambda^{\pm} = \frac{(A+1-1/\tau) \pm \sqrt{\Delta}}{2}\,, \tag{37}$$

$$\Delta = (A+1-1/\tau)^2 - 4(1-1/\tau)A = [A-(1-1/\tau)]^2\,. \tag{38}$$

Notice that $\Delta \geq 0$ so the eigenvalues are always real: the fixed point is an attractive or repulsive node. The final solution of Eq. 37 is:

$$\lambda^+ = A\,, \tag{39}$$

$$\lambda^- = 1 - 1/\tau\,. \tag{40}$$

This means that the fixed point $(\rho^0 = 0, \Gamma^0 = A)$ is stable for $\lambda^+ = A < 1$. The critical point is $A = 1$.

This analysis show that we can have a subcritical state $(0, A)$, an attractive node, if we use $A < 1$. On the other hand, if we use $A > 1$, the absorbing state looses its stability and the non trivial fixed point (ρ^*, Γ^*) is the unique stable solution. For $A = 1$, the point $(\rho^0 = 0, \Gamma^0 = 1)$ is an indifferent node.

Evaluating the derivatives at the non trivial fixed point, we get:

$$\left.\frac{\partial F}{\partial \rho}\right]_* = \frac{3 - A + u\tau}{A + u\tau + 1}\,, \tag{41}$$

$$\left.\frac{\partial F}{\partial \Gamma}\right]_* = \frac{(A-1)(2+u\tau)^2}{(A+u\tau+1)(2A+u\tau)^2}\,, \tag{42}$$

$$\left.\frac{\partial G}{\partial \rho}\right]_* = -\frac{u(2A+u\tau)}{2+u\tau}\,, \tag{43}$$

$$\left.\frac{\partial G}{\partial \Gamma}\right]_* = 1 - \frac{1}{\tau} - \frac{u(A-1)}{2A+u\tau}\,, \tag{44}$$

As before, the eigenvalues of the Jacobian matrix at the fixed point (ρ^*, Γ^*) are determined by Eq. (8). After some algebra, we get:

$$\begin{aligned} |\lambda^+\lambda^-| &= ad - bc \\ &= \frac{(1 - 1/\tau - \frac{u(A-1)}{2A+u\tau})(3 - A + u\tau)}{(A+u\tau+1)} + \frac{u(2+u\tau)(A-1)}{(2A+u\tau)(A+u\tau+1)}\,, \\ &= \left(1 - \frac{1}{\tau}\right)\left(1 - \frac{2(A-1)}{A+u\tau+1}\right) + \frac{u(A-1)^2}{(A+u\tau+1)(2A+u\tau)}\,. \end{aligned} \tag{45}$$

For large τ, we have:

$$|\lambda^{\pm}| = \sqrt{\lambda^+\lambda^-} = 1 - \frac{2(A-1)+u}{2u\tau} + O(\tau^{-2})\,, \tag{46}$$

which means that, for large τ, the map is very close to a Neimark-Sacker-like critical point. As before, the focus can be excited by fluctuations, generating the SO.

The frequency for small amplitude oscillations is $\omega \equiv |\omega^{\pm}| = \arctan\sqrt{\frac{4D}{T^2}-1}$, where D and T are respectively the determinant and trace of the Jacobian matrix. When $\tau \to \infty$,

$$\omega \simeq \sqrt{\frac{A-1}{\tau}} \tag{47}$$

which is similar to the $\tau^{-1/2}$ behavior of the simple gain dynamics, Eq. (16).

Stability analysis of the Cellular Automata model with LHG synapses

Starting from Eqs (24 and 25) of the main Manuscript, the 2d MF map close to the stationary state is:

$$\rho[t+1] = (1-\rho[t])\left[1-\left(1-\frac{\sigma[t]\rho[t]}{K}\right)^K\right] = F(\rho[t],\sigma[t])\,, \tag{48}$$

$$\sigma[t+1] = \sigma[t]+\frac{1}{\tau}(A-\sigma[t]) - u\sigma[t]\rho[t] = G(\rho[t],\sigma[t])\,, \tag{49}$$

As before, the stability of the map is given by the eigenvalues of the Jacobian matrix $J(\rho,\sigma)$ at the fixed points. The matrix elements are:

$$\left.\frac{\partial F}{\partial \rho}\right] = \left[\left(1-\frac{\sigma\rho}{K}\right)^K - 1\right] + (1-\rho)\sigma\left(1-\frac{\sigma\rho}{K}\right)^{K-1}, \tag{50}$$

$$\left.\frac{\partial F}{\partial \sigma}\right] = (1-\rho)\rho\left(1-\frac{\rho\sigma}{K}\right)^{K-1}, \tag{51}$$

$$\left.\frac{\partial G}{\partial \rho}\right] = -u\sigma\,, \tag{52}$$

$$\left.\frac{\partial G}{\partial \sigma}\right] = 1-\frac{1}{\tau}-u\rho\,. \tag{53}$$

Thus, we can plug in the solutions of Eqs (26 and 27) of the main Manuscript in Eqs (50-53) and find the eigenvalues of $J(\rho,\sigma)$.

Similarly to the previous model, the MF map of the CA with LHG synapses has two fixed points. The first one is the absorbing state fixed point and is given by

$$\rho^0 = 0 \qquad \sigma^0 = A\,. \tag{54}$$

The second one is nontrivial and must be solved numerically.

We can compute the elements of the Jacobian matrix at the absorbing state fixed point

$$\left.\frac{\partial F}{\partial \rho}\right]_0 = A\,, \tag{55}$$

$$\left.\frac{\partial F}{\partial \sigma}\right]_0 = 0\,, \tag{56}$$

$$\left.\frac{\partial G}{\partial \rho}\right]_0 = -uA\,, \tag{57}$$

$$\left.\frac{\partial G}{\partial \sigma}\right]_0 = 1-\frac{1}{\tau}\,, \tag{58}$$

which is the same result of Eqs (31–34). This matrix has two eigenvalues: $1-1/\tau$ and A. The eigenvalue $1-\tau$ corresponds to the σ axis, which is a stable direction. The eigenvalue A corresponds to a unstable (stable) direction for $A>1$ ($A<1$).

If we assume that we are close to criticality, where ρ^* is small, we can find an approximate solution for $J(\rho,\sigma)$ at the nontrivial fixed point. Expanding the binomial in Eq. (24) of the main Manuscript to first order and solving for ρ^*, we find:

$$\rho* = \frac{A-1}{A+u\tau}\,, \tag{59}$$

$$\sigma^* = \frac{A+u\tau}{1+u\tau} = 1+\frac{A-1}{1+u\tau}\,, \tag{60}$$

where we used Eq. (27) of the main Manuscript to compute σ^*. Notice that, as before, this solution exists only if $A-1>0$. This is the exact condition to the absorbing state solution to be unstable.

Inserting in Eqs (50-53), we have:

$$J = \begin{pmatrix} 1-a & b \\ -c & 1-d \end{pmatrix}, \tag{61}$$

where

$$a = \frac{(2K-1)(A-1)}{K(1+u\tau)}, \tag{62}$$

$$b = \frac{A-1}{(A+u\tau)^2}\left(1+u\tau-\frac{(A-1)(K-1)}{K}\right), \tag{63}$$

$$c = \frac{u(A+u\tau)}{1+u\tau}, \tag{64}$$

$$d = \frac{1}{\tau}+\frac{u(A-1)}{A+u\tau}. \tag{65}$$

Note that they are all positive quantities. The eigenvalues of J are:

$$\lambda^{\pm} = 1-\frac{a+d}{2}\pm\frac{1}{2}\sqrt{(a-d)^2-4bc}. \tag{66}$$

For systems to be close to criticality, we must have $\tau \gg 1$. Thus, a, b and d are of $O(1/\tau)$, while $c = O(1)$. Therefore, the $4bc$ term dominates the square root argument in Eq. (66). Hence, we have two complex conjugate eigenvalues with modulus and argument given by:

$$|\lambda^{\pm}| = \sqrt{1-(a+d)+da+bc}. \tag{67}$$

We can expand $|\lambda^{\pm}|$ in powers of $1/\tau$:

$$|\lambda^{\pm}| = 1-\left(\frac{(A-1)(2K-1)}{2uK}+\frac{1}{2}\right)\frac{1}{\tau}+O(\tau^{-2}). \tag{68}$$

This confirms that the same scenario of weakly stable focus appears in the CA model. The harmonic frequency is:

$$\omega \equiv |\omega^{\pm}| = \arctan\frac{\sqrt{4bc-(a-d)^2}}{2-(a+d)}. \tag{69}$$

To first order in τ, we get $\omega \simeq \sqrt{(A-1)/\tau}$, which shows that the frequency of oscillations is vanishingly small for large τ, being identical to Eq. (47).

References

1. Brochini, L. *et al.* Phase transitions and self-organized criticality in networks of stochastic spiking neurons. *Sci. Rep.* **6**, 35831 (2016).
2. Costa, A. A., Brochini, L. & Kinouchi, O. Self-organized supercriticality and oscillations in networks of stochastic spiking neurons. *Entropy* **19**, 399 (2017).
